# Prevalence and predictors of peripherally inserted central catheter-associated bloodstream infections in adults: A multicenter cohort study

**DOI:** 10.1371/journal.pone.0213555

**Published:** 2019-03-07

**Authors:** Jae Hwan Lee, Eung Tae Kim, Dong Jae Shim, Il Jung Kim, Jong Hyun Byeon, In Joon Lee, Hyun Beom Kim, Young Ju Choi, Jin Hong Lee

**Affiliations:** 1 Center for Liver Cancer, National Cancer Center, Goyang-si, Gyeonggi-do, Republic of Korea; 2 National Cancer Center, Goyang-si, Gyeonggi-do, Republic of Korea; 3 Department of Radiology, Hanyang University Guri Hospital, Guri-si, Gyeonggi-do, Republic of Korea; 4 Department of Radiology, College of Medicine, Kangwon National University, Chuncheon-si, Gangwon-do, Republic of Korea; 5 Department of Radiology, Incheon St. Mary's Hospital, College of Medicine, The Catholic University of Korea, Seoul, Republic of Korea; 6 Department of Radiology, Bucheon St. Mary's Hospital, College of Medicine, The Catholic University of Korea, Seoul, Republic of Korea; University of Florida, UNITED STATES

## Abstract

**Objective:**

To evaluate the prevalence and predictors of peripherally inserted central catheter-associated bloodstream infection (PBSI) and PBSI-related death in hospitalized adult patients.

**Materials and methods:**

A retrospective multicenter cohort of consecutive patients who underwent PICC placement from October 2016 to September 2017 at four institutes was assembled. Using multivariable logistic and Cox-proportional hazards regression models, all risk factors were analyzed for their association with PBSI. Multivariable logistic models were used to evaluate predictors of PBSI-related death.

**Results:**

During the study period, a total of 929 PICCs were inserted in 746 patients for a total of 17,913 catheter days. PBSI occurred in 58 patients (6.2%), with an infection rate of 3.23 per 1,000 catheter days. Number of catheter lumens [double lumen, odds ratio (OR) 5.295; 95% confidence interval (CI), 2.220–12.627; hazard ration (HR) 3.569; 95% CI, 1.461–8.717], PICC for chemotherapy (OR 4.94; 95% CI, 1.686–14.458; HR 7.635; 95% CI, 2.775–21.007), and hospital length of stay (OR 2.23; 95% CI, 1.234–4.049; HR 1.249; 95% CI, 0.659–2.368) were associated with PBSI. Risk factors, such as receiving chemotherapy (OR 54.911; 95% CI, 2.755–1094.326), presence of diabetes (OR 11.712; 95% CI, 1.513–90.665), and advanced age (OR 1.116; 95% CI 1.007–1.238), were correlated with PBSI-related death.

**Conclusion:**

Our results indicated that risk factors associated with PBSI included the number of catheter lumens, the use of PICCs for chemotherapy, and the hospital length of stay. Furthermore, PBSI-related death was common in patients undergoing chemotherapy, diabetics, and elderly patients.

## Introduction

Peripherally inserted central catheters (PICCs) are increasingly used in contemporary medicine because of their characteristics of feasibility, accessibility, safety, versatility, and cost-effectiveness [1]. The growing use of PICCs also stems from the seeming superiority of PICCs to other central venous catheters with respect to risk of hospital-acquired bloodstream infection, which is an important and preventable cause of the morbidity and mortality in hospitalized patients [2, 3]. Possible reasons suggested for this lower risk of infection include the lower bacterial density and lower temperature of the PICC placement site compared with neck or groin placement sites of other central venous catheters [4]. However, recent data from hospitalized patients suggest that PICC-associated blood stream infection (PBSI) rates vary among different patient settings and are actually comparable to blood stream infection rates of standard central venous catheters [1, 4, 5]. Other studies reveal that the PBSI rate is not lower than the central line-associated bloodstream infection (CLABSI) rate, which ranges from 0.6 to 7.4% for catheter days ranging from 0.07 to 2.46 per 1000 days [1, 3, 6–9]. These studies also show that the occurrence of CLABSI is more frequent in patients with intensive care unit (ICU) stays and patients with hematologic malignancies [1, 6–9]. These varying data raise the question of whether PICCs are truly safer than central venous catheters with respect to catheter-associated blood stream infections. Despite several reports regarding risk factors of PBSI in single centers or among oncologic patients [6–8], there has been no investigation evaluating prevalence and predictors of PBSI in varied patient care environments that reflect the real-world situation. Given the important role of CLABSI in patient mortality [10], there is a surprising paucity of data to specifically identify predictors of PBSI-related death. To better inform clinicians regarding PICC use and improve patient safety, the factors associated with adverse clinical outcome must be elucidated. Consequently, the purpose of this study was to evaluate the prevalence and predictors of PBSI and PBSI-related death in hospitalized adult patients.

## Materials and methods

### Study design and data collection

**Fig 1:**
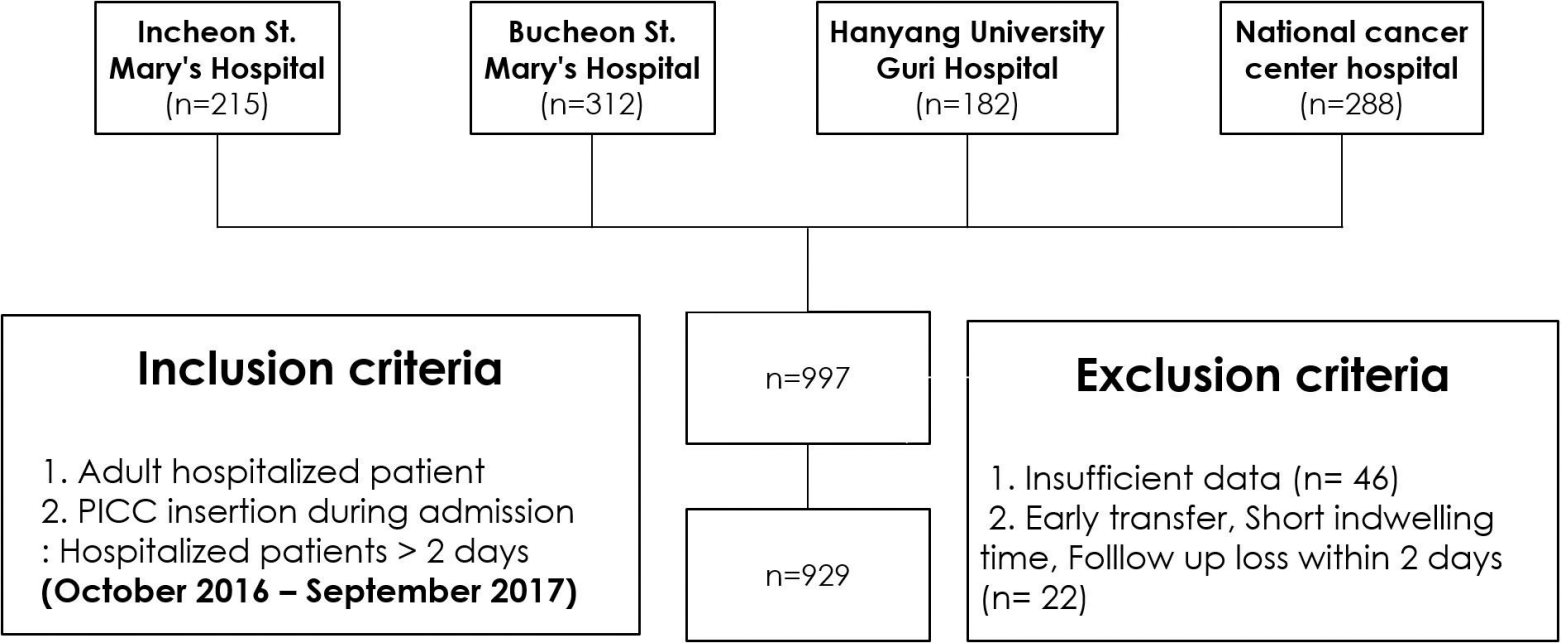
Study enrollment.

This was a multicenter retrospective cohort study conducted in four hospitals, including two tertiary institutions, a single secondary institution, and a national cancer center hospital. Briefly, Incheon St. Mary's Hospital and Bucheon St. Mary's Hospital are 901- and 613-bed tertiary general hospital, respectively. Hanyang University Guri Hospital are 578-bed secondary general hospital respectively. National cancer center hospital are 555-bed secondary cancer hospital respectively. All participating hospitals are teaching hospitals and located in urban area. The institutional review boards of each site (CMC-IRB, NCC-IRB, HY Guri-IRB) approved this study, and informed patient consent was waived. Medical records of consecutive adult inpatients receiving PICC between October 2016 and September 2017 at each site were collected. Patients were excluded if they transferred early, had short indwelling times (<2 days), or lacked information regarding the insertion or removal date of PICC (Fig 1). Therefore, hospitalized patients more than 2 days were included.

### PICC insertion and management

All participating institutions followed the Korean Nosocomial Infection Surveillance and Prevention Protocol [11] and United States guidelines for preventing catheter-related infections [12]. In brief, maximal sterile barrier precautions with skin decontamination using iodine tincture or 2% chlorhexidine gluconate were applied before PICC placement. Single or dual 4–6 French (Fr) lumen catheters (PowerPICC, Bard Access Systems Inc., Salt Lake City, UT, USA; Turbo-Ject PICC, Cook Medical, Bloomington, IN, USA; Pro-PICC, Medical Components Inc., Harleysville, PA, USA) were used in our study. All PICCs were inserted by trained interventional radiologists who followed the protocol of using the smallest caliber of PICC in the largest vein available. The procedure was performed in a dedicated angiography suite or at bedside in cases of some ICU patients. Each procedure was done using ultrasonography and fluoroscopy guidance, followed a maximal evidence-based institutional aseptic protocol. All percutaneous access was performed with ultrasonography at the upper arm. The upper arm was the preferred location to insert PICCs unless there were clinical contraindications (e.g., previous axillary operation or radiotherapy, arm edema, or arteriovenous fistula for dialysis access). At the end of the procedure, the catheter tip location was assessed by fluoroscopy or plain radiography to determine if it was placed at the cavoatrial junction. Regular device checks took place according to the protocol of each institution. Insertion-site care entailed weekly changes of transparent, semi-permeable film-covered dressing and new sterile gauze application every other day in all hospitals.

### Definition and variables

The National Healthcare Safety Network surveillance definition of PBSI was used [13]. In brief, PBSI is a primary bloodstream infection in a patient who has had a PICC in place for >2 days and has a recognized pathogen (identified from one or more blood specimens by a culture- or non-culture-based microbiologic test) that is not related to an infection at another site. If laboratory studies failed to identify causative bacteria, the case was not identified as PBSI to reduce potential selection bias among different hospital environments. The prevalence of PBSI was calculated as percentage and rate per catheter day [8].

A patient death was defined as related to PBSI when systemic bacteremia from a PBSI existed prior to the patient’s death and the cause of death was assumed to be due to the progression of PBSI-related sepsis [10]. Death certificates of all cases of deceased patients were reviewed to identify the cause of death. Patients with other causes of death, such as aggravation of underlying disease or infection other than PBSI, were excluded. All medical records of potential PBSI or PBSI-related death cases were reviewed manually to confirm whether they met the PBSI definition.

Variables regarding PICC use were analyzed using a conceptual model of predictors of PICC complication [7]. In brief, this model consists of patient, device, and provider factors that could influence PICC-related complications. The ICU status was defined as patients who required any ICU care during hospitalization. The indication of PICC placement was based on initial purpose of catheterization, such as venous access and hydration, parenteral nutrition, antibiotics delivery, or chemotherapy. Dwell time of PICCs was calculated in days by subtracting the date of removal from the date of PICC insertion. Two reviewers adjudicated these data to ensure agreement on assignment.

### Statistical analysis

The unit of all statistical analyses was each PICC insertion. Mixed-effects binary logistic regression was used to predict PBSI and PBSI-related death, adjusting for patient-, device-, and provider-level characteristics. Predictors of PBSI and PBSI-related death were evaluated first by univariable tests, and then by using a full multivariable model including variables with *p* values < 0.20. Cox proportional hazards regression was used to estimate adjusted hazard ratios (aHRs) and 95% confidence intervals (CIs) for ‘time to infection’ of PBSI. Statistical significance was set at *p<*0.05. SPSS for Windows (v18.0; SPSS Inc., Chicago, IL, USA) and Stata MP SE (StataCorp, College Station, TX, USA) were used for analyses.

## Results

Between October 2016 and September 2017, 929 PICCs were placed in 746 individual patients, resulting 17,913 catheter days. Nearly half of the patients had malignant solid tumors (n = 514; 55.4%). The most common indications for PICC insertion were intravenous infusion (56.6%; n = 526), antibiotics therapy (23.5%; n = 218), total parenteral nutrition (TPN; 16.0%; n = 149), and delivery of chemotherapy (3.8%; n = 35). Almost two thirds of catheters were double-lumen devices (62.7%; n = 592), which represents a device-related factor. With respect to provider characteristics, the majority of catheters were placed in the patient’s right upper arm (65.7%, n = 610; Table 1).

**Table 1:** Descriptive statistics for patient, device, and provider predictors of PBSI according to PBSI status.

Predictor	Total (n = 929)	Non-PBSI (n = 871)	PBSI (n = 58)	*p* value
**Patient-Related**				
Mean age ± SD (yr)	66.6±15.5	66.5±15.5	67.8±15.1	0.511
Gender (Male)	428 (46.1)	400 (45.9)	28 (48.3)	0.068
Comorbidities				
None	246 (26.5)	230 (26.4)	16 (27.6)	Ref
Diabetes mellitus (type II)	141 (15.2)	127 (14.6)	14 (24.1)	0.177
Malignant solid tumor	514 (55.3)	487 (55.9)	26 (44.8)	0.416
Hematologic malignancy or neutropenia (ANC<500)	36 (3.7)	34 (3.9)	2 (3.4)0	0.858
Multicomorbidity (≥2)	7 (0.7)	7 (0.7)	0 (0.0)	-
Intensive care unit stay (days)	134 (14.4)	122 (14.0)	12 (20.7)	0.175
Presence of additional intravascular device	52 (5.6)	47 (5.4)	5 (8.6)	0.367
Hospital length of stay (days)	45.9 [2–839]^†^	44.9 [2–839]^†^	61.4 [13–210]^†^	0.073
Indication				
Intravenous infusion	526 (56.6)	501 (57.5)	25 (43.1)	Ref
TPN	149 (16.0)	141 (16.2)	7 (12.1)	0.991
Antibiotics therapy	218 (23.5)	197 (22.6)	21 (36.2)	0.014
Chemotherapy	35 (3.8)	31 (3.4)	5 (8.6)	0.022
Dwell time (days)	20.4 [2–239] ^†^	20.3 [2–239]*	23.5 [3–84]^†^	0.006
**Device-Related**				
Lumens				
Single	337 (36.3)	331 (38.0)	6 (10.3)	Ref
Double	592 (62.7)	540 (62.0)	52 (89.7)	<0.001
**Provider-Related**				
Arm				
Right	610 (65.7)	564 (64.8)	46 (79.3)	0.027
Vein				
Basilic	616 (66.3)	576 (66.1)	40 (69.0)	Ref
Brachial	257 (27.7)	242 (27.8)	15 (25.9)	0.716
Cephalic	56 (6.0)	53 (6.1)	3 (5.2)	0.740

Note: Unless otherwise indicated, data are expressed as numbers of patients. Numbers in parenthesis are percentages. Key: (PBSI) peripherally inserted central catheter-associated bloodstream infection, (ANC) absolute neutrophil count; (TPN) total parenteral nutrition

(*) mean [range].

Among 929 PICCs evaluated in our analysis, 58 (6.2%) developed PBSI over 1366 catheter days, corresponding to 3.23 per 1000 catheter days. With respect to microbiology, coagulase negative *staphylococci* (44.8%), *staphylococcus aureus* (17.2%), *Candida* species (6.9%), and *Escherichia coli* (6.8%) account for most of the causative bacteria (Table 2).

**Table 2:** Microbiology of PBSI.

Pathogen	Number of infections (n = 58) (%)
Gram-positive bacteria Coagulase-negative staphylococci *Staphylococcus aureus* (MRSA + MSSA) *Enterococcus species* *Micrococcus species* *Clostridium perfringens*	41 (70.7) 26 (44.8) 10 (17.2) 2 (3.4) 2 (3.4) 1 (1.7)
Gram-negative bacteria *Escherichia coli* *Acinetobacter baumannii* *Enterobacter cloacae* *Klebsiella pneumoniae* *Burkholderia cepacia*	13 (22.4) 4 (6.8) 4 (6.8) 2 (3.4) 2 (3.4) 1 (1.7)
Candida species	6 (6.9)
*Candida albicans* *Candida tropicalis*	4 (6.8) 2 (3.4)
Polymicrobial infections	2 (3.4)

Key: (PBSI) peripherally inserted central catheter-associated bloodstream infection; (MRSA) Methicillin-resistant *Staphylococcus aureus*; (MSSA) methicillin-susceptible *Staphylococcus aureus*

Univariable analysis indicated PBSI was associated with patients who had PICC placement in the left arm for antibiotic therapy and with chemotherapy. Importantly, PBSI was strongly associated with number of catheter lumens, with double lumen catheters presenting a greater risk than a single lumen catheter. Multivariable model analysis revealed that patients receiving PICCs for chemotherapy, hospital length of stay, and the number of catheter lumens were significant predictors for PBSI (Table 3). Specifically, PICCs with double lumens showed greater risk (OR 5.295; 95% CI, 2.220–12.627) than the other predictors. The HRs of patients receiving PICCs for chemotherapy were higher than the ORs, suggesting that an indication for chemotherapy was associated not only with PBSI but also earlier time to infection (OR 4.937; 95% CI 1.686–14.458, HR 7.635; 95% CI 2.775–21.007).

**Table 3:** Multivariable logistic and Cox proportional hazards regression models of predictors of PBSI.

Predictor	OR (95% CI)	*p* value	aHR (95% CI)	*p* value
**Patient-related**				
Gender (Male/Female)	1.084 (0.623–1.885)	0.775	1.068 (0.627–1.818)	0.810
Diabetes mellitus (type II)	1.325 (0.670–2.620)	0.418	1.284 (0.670–2.460)	0.451
Intensive care unit stay (d)	1.087 (0.544–2.170)	0.813	1.142 (0.593–2.200)	0.691
Indication				
Intravenous infusion	1 (ref)	Ref	1 (ref)	Ref
TPN	1.205 (0.503–2.889)	0.676	1.239 (0.527–2.96)	0.623
Antibiotics	1.722 (0.927–3.198)	0.085	1.669 (0.909–3.066)	0.099
Chemotherapy	4.937 (1.686–14.458)	0.004	7.635 (2.775–21.007)	<0.001
Hospital length of stay (d)	2.235 (1.234–4.049)	0.008	1.249 (0.659–2.368)	0.496
Dwell time (d)	1.364 (0.749–2.487)	0.310	N/A	N/A
**Device-related**				
Lumens (Double/single)	5.295 (2.220–12.627)	<0.001	3.569 (1.461–8.717)	0.005
**Provider-related**				
Arm (Left/Right)	1.356 (0.671–2.742)	0.396	1.341 (0.680–2.644)	0.398

Key: (PICC) peripherally inserted central catheter; (OR) odds ratio; (aHR) adjusted hazard ratio; (CI) confidence interval; (TPN) total parenteral nutrition; (ICU) intensive care unit; (N/A) not applicable.

A total of 45 patients died during the study period. Among them, six patients (13.4%) were confirmed as having a PBSI-related death. Univariable and multivariable analyses for predictors of PBSI-related death showed that use of PICCs for chemotherapy, type II diabetes, and advanced age were factors associated with PBSI-related death (Table 4). The mean age of patients who suffered PBSI-related death was significantly older than that of the other patients (79.67±4.80; 95% CI 74.63–84.71 vs. 66.50±15.47; 95% CI 65.50–67.50, p = 0.039). We found no association between device or provider factors and risk of PBSI-related death.

**Table 4:** Univariable and multivariable logistic regression analysis of predictors of PBSI-related death.

Predictor	Univariable analysis		Multivariable analysis	
	OR (95% CI)	*p* value	OR (95% CI)	*p* value
Patient-Related				
Mean age	1.091 (1.004–1.185)	0.039	1.115 (1.006–1.235)	0.038
Gender (Male/Female)	1.172 (0.235–5.836)	0.847		
Comorbidities				
None	1 (ref.)	0.995		
Diabetes mellitus (type II)	11.474 (2.081–63.255)	0.005	11.828 (1.537–91.030)	0.018
Malignant solid tumor	2.491 (0.454–13.670)	0.293		
Hematologic malignancy or neutropenia (ANC<500)	1.092 (0.000–1.007)	0.998		
Intensive care unit stay (d)	2.996 (0.543–16.522)	0.208		
Presence of additional intravascular device	1.092 (0.000–1.011)	0.998		
Hospital length of stay (d)	0.999 (0.985–1.013)	0.916		
Indication				
Intravenous infusion or TPN	1 (ref.)	Ref.	1 (ref.)	Ref.
Antibiotics therapy	4.667 (0.775–28.112)	0.159	2.621 (0.405–16.950)	0.312
Chemotherapy	9.882 (0.874–111.699)	0.064	68.044 (3.426–1351.444)	0.006
Dwell time (d)	0.985 (0.932–1.041)	0.596		
Device-Related				
Lumens (double/single)	1.139 (0.208–6.254)	0.881		
Provider-Related				
Arm (right/left)	0.000	0.994		
Vein				
Basilic	1 (ref.)	0.797		
Brachial	0.477 (0.055–4.106)	0.501		
Cephalic	0.000	0.998		

Key: (PBSI) peripherally inserted central catheter-associated blood stream infection; (OR) odds ratio; (CI) confidence interval; (ANC) absolute neutrophil count; (TPN) total parenteral nutrition; (N/A) not applicable.

## Discussion

In this study, PBSI occurred in 6.2% of patients with total PICC insertion, resulting in an infection rate of 3.23 per 1,000 catheter days. This PBSI rate is higher than that previously reported in single-center studies, which may be due to the heterogeneous patient groups and patient-care environments in our multicenter cohort. One of the hospitals in the multicenter cohort is a national cancer center whose patients primarily had active cancer, and more than half of the patients in our study population had a solid or hematologic malignancy. Moreover, our study only enrolled hospitalized patients who were by definition more susceptible to hospital-acquired infections than outpatients. These results provided a more realistic overview of PICC management in daily practice.

Our results also showed that the use of PICC for chemotherapy was not only associated with PBSI but may also accelerate the development of PBSI. Previous reports show that chemotherapy plays an important role in PBSI occurrence in hospitalized patients, probably due to immunosuppression by various chemotherapy drugs that make the patient vulnerable to infection [8]. These reports emphasize the importance of PICC management in susceptible patients. Interestingly, in contrast to a previous study (6), PBSI was not associated with patients with hematologic malignancy or neutropenia in our study. This discrepancy might be due to the very low patient population (n = 36, 3.7%) of hematologic malignancy or neutropenia in our study, which limited statistical evaluation.

Increasing the number of the catheter lumen was strongly associated with the development of PBSI. This result is similar to a previous study that found an increasing order of PBSI risk associated with double- to triple-lumen catheters (HR 4.08 and 8.52, respectively) [7]. A possible explanation for these results might be that increased manipulation of the catheter hub and catheter surface area augments bacterial translocation and migration to systemic blood circulation.

Consistent with a previous study [7], our results showed that hospital length of stay, rather than the catheter dwell time, increased the risk of PBSI. We speculate that a longer hospital stay reflects the severity of a patient’s illness and increases the chance of hospital-acquired infections.

PBSI-related death was associated with old age, diabetes, and receiving chemotherapy. Although a previous study [10] suggests that CLABSI is associated with an increased risk of death, there has been a paucity of data to directly reveal predictors of PBSI-related death. Interestingly, the prevalence of PBSI was strongly associated with device factor, whereas risk of PBSI-related death was associated only with patient factor. We postulate that when PBSI occurs, susceptible patients are at an increased risk of death. This explanation supports the need for meticulous care for patients with PICCs, such as using single rather than multiple lumen catheters in high-risk patients to decrease the incidence of PBSI and PBSI-associated mortality.

The present study has several limitations. First, the retrospective design of our study is subject to selection bias. To minimize bias and enhance generalizability, we performed concurrent analyses of all consecutive patients at the different multicenter settings. Second, all PICC insertions in our study were performed by interventional radiologists. A previous study has suggested that a provider factor can influence the clinical outcome of PBSI [7]. However, we were unable to test that aspect because of a lack of venous access nursing teams to perform PICC insertions in the participating institutions. Third, we did not find any association between catheter dwell time and PBSI, possibly because PICCs were often removed without microbiological confirmation when PICC-related infection was clinically suspected. Fourth, there was a relatively small number of PICC-related death in this study, so that statistical significance of variables regarding PBSI-related death might be exaggerated. However old age, the presence of diabetes, and PICC use for chemotherapy could be regarded as clinically relevant factors to explain PBSI-related death. Further study with prospective design is needed for precise evaluation of PBSI-related death.

In conclusion, PBSI was associated with the number of catheter lumens, the use of PICC for chemotherapy, and the hospital length of stay. PBSI-related deaths were common in patients receiving chemotherapy, type II diabetics, and elderly patients. Full data set of present study is on S1 Table.

## Supporting information

S1 Table Full data set of present study. (XLSX)
